# Addressing multilevel barriers to clinical trial participation among Black and White men with prostate cancer through the PACCT study

**DOI:** 10.1002/cam4.5552

**Published:** 2022-12-20

**Authors:** Susan Eggly, Nicole Senft, Seongho Kim, Elisabeth I. Heath, Hyejeong Jang, Tanina F. Moore, Fatmeh Baidoun, Mark A. Manning, Louis A. Penner, Terrance L. Albrecht, Michael A. Carducci, Dina Lansey, Lauren M. Hamel

**Affiliations:** ^1^ Department of Oncology Wayne State University/Karmanos Cancer Institute Detroit Michigan USA; ^2^ Department of Psychology Oakland University Rochester Michigan USA; ^3^ Johns Hopkins Sidney Kimmel Comprehensive Cancer Center Baltimore Maryland USA

**Keywords:** clinical trials, health disparities, patient–physician communication, prostate cancer, question prompt list

## Abstract

**Background:**

Cancer clinical trial participation is low and inequitable. Partnering Around Cancer Clinical Trials (PACCT) addressed systemic and interpersonal barriers through an observational study of eligibility and an intervention to improve patient–physician communication and trial invitation rates.

**Methods:**

Physicians at two comprehensive cancer centers and Black and White men with prostate cancer participated. Patients were followed for 2 years to determine whether they became potentially eligible for an available therapeutic trial. Potentially eligible patients were randomized to receive a trials‐focused Question Prompt List or usual care. Patient–physician interactions were video‐recorded. Outcomes included communication quality and trial invitation rates. Descriptive analyses assessed associations between sociodemographic characteristics and eligibility and effects of the intervention on outcomes.

**Results:**

Only 44 (22.1%) of participating patients (*n* = 199) became potentially eligible for an available clinical trial. Patients with higher incomes were more often eligible (>$80,000 vs. <$40,000, adjusted OR = 6.06 [SD, 1.97]; $40,000–$79,000 vs. <$40,000, adjusted OR = 4.40 [SD, 1.81]). Among eligible patients randomized to the intervention (*n* = 19) or usual care (*n* = 25), Black patients randomized to the intervention reported participating more actively than usual care patients, while White intervention patients reported participating less actively (difference, 0.41 vs. −0.34). Intervention patients received more trial invitations than usual care patients (73.7% vs. 60.0%); this effect was greater for Black (80.0% vs. 30.0%) than White patients (80.0% vs. 66.7%).

**Conclusions:**

Findings suggest the greatest enrollment barrier is eligibility for an available trial, but a communication intervention can improve communication quality and trial invitation rates, especially for eligible Black patients.

## Graphical abstract

This multisite study addressed two barriers to enrollment of Black and White men in prostate cancer clinical trials—patient eligibility and patient–physician communication. Findings showed eligibility, a systemic barrier, is the biggest barrier; but a simple Question Prompt List communication intervention can improve interpersonal barriers including communication quality and trial invitation rates, especially for Black patients.

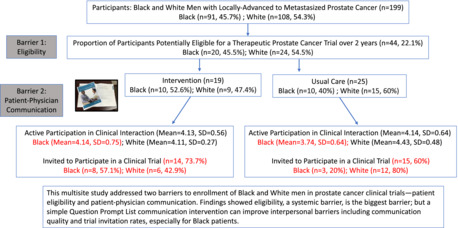

## 1 INTRODUCTION

Cancer clinical trials test the safety and efficacy of promising treatments and translate new knowledge into tangible benefits for patients. However, only a small percentage of patients participate.^1^ Low accrual jeopardizes assessment of the safety and effectiveness of new approaches to cancer care, waste resources, and precludes follow‐up studies.

Despite NIH and other professional society requirements to include people of color in clinical research,^2^, ^3^ under‐enrollment is an even greater problem in these groups, especially among Black people.^4^, ^5^, ^6^, ^7^, ^8^ Inequitable enrollment limits the generalizability of findings^7^, ^9^ and may represent inequitable access to high‐quality clinical care.^10^

This problem is especially clear in prostate cancer, which is estimated to account for 27% of all cancer diagnoses and 11% of deaths in men in 2022.^11^ Black men are 1.73 times more likely to be diagnosed and 2.13 times more likely to die from prostate cancer than non‐Hispanic White men.^12^ Yet, accrual to prostate cancer clinical trials among Black men is disproportionately low,^13^, ^14^ including among men with metastatic disease.^15^

While enrollment barriers are complex and multilevel,^16^ research demonstrates that most patients are willing to participate, with little difference by race,^17^, ^18^, ^19^ suggesting causes beyond individual preference. In this research, we examine two additional enrollment barriers that may disproportionately affect Black patients, one systemic and one interpersonal: eligibility for an available trial and patient–physician communication. We report on the first phase of a prospective, multi‐site, multilevel study, Partnering Around Cancer Clinical Trials (PACCT). Designed to better understand and improve participation in prostate cancer clinical trials, especially among Black men, PACCT included a prospective observational study examining eligibility, and, for a subset of participants, a prospective communication intervention study.

The objective of the prospective observational study was to describe the proportion and socio‐demographic characteristics of patient participants who preliminarily met eligibility criteria for an available prostate cancer therapeutic clinical trial during the study period. We expected that sociodemographic characteristics, such as self‐identified race, age, income, and education, as well as self‐reported health status would be associated with likelihood of potential eligibility.^4^, ^20^

The prospective interventional study focused on patient–physician clinical communication among the subset of patients identified as potentially eligible for an available trial. The objective was to examine whether a patient‐level communication intervention improved communication quality and rates of invitations to participate in an available prostate cancer therapeutic clinical trial. We expected that patients who received the intervention, compared to patients in the usual care group, would experience better communication quality and be more likely to receive an invitation. We further expected that intervention effects would be greater for Black than for White participants, given documented disparities in communication quality during interactions with Black and with White patients.^21^

## 2 METHODS

### 2.1 Study design

Study design included a prospective descriptive study and a prospective randomized clinical trial. In the original protocol,^22^ PACCT was a multi‐site, multi‐level study with two phases, each involving an independent communication intervention. The first phase, reported here, included a randomized clinical trial of a patient‐focused communication intervention. The second phase involved a physician‐focused communication training intervention that provided patient‐centered communication strategies and encouragement to discuss trials with all potentially eligible patients, regardless of race or other sociodemographic circumstances, using high‐quality, patient‐centered communication. Here we report on data from Phase 1, because, although the physician intervention was implemented and found to be acceptable and effective,^23^ data collection for Phase 2 was disrupted by the COVID pandemic and could not be completed.^24^, ^25^ This disruption also led to some adjustments in both outcome measures and analysis, described below.

### 2.2 Settings and participants

PACCT was conducted at two National Cancer Institute‐designated comprehensive cancer centers: Wayne State University/Karmanos Cancer Institute (WSU/KCI) in Detroit, Michigan, and Johns Hopkins/Sidney Kimmel Comprehensive Cancer Center (SKCCC) in Baltimore, Maryland. Physicians (medical oncologists, urologists, and radiation oncologists) were eligible to participate if they treated patients with prostate cancer and could recruit patients to available trials. Adult men were eligible if they had a confirmed diagnosis of prostate cancer (locally‐advanced to metastatic); self‐identified as Black, African American, or White and non‐Hispanic; had an ongoing but relatively brief (<1 year) relationship with a participating physician and expected to see the physician at least once in the following year; could read and write English; and could potentially qualify for a therapeutic clinical trial within 2 years of consent (e.g., good performance status, no significant comorbidities). Although patients were technically eligible to participate if they had a confirmed diagnosis of prostate cancer, recruitment of patients with intermediate‐ to high‐risk prostate cancer was prioritized because they were more likely to become eligible for a therapeutic clinical trial than patients with early‐stage, low‐risk disease. Any open prostate cancer trial was appropriate, spanning from peri‐operative, radiation‐based, or drug therapy for recurrent or metastatic disease. The study was approved by the institutional review boards at each site and registered at Clinicaltrials.gov (identifier NCT02906241). All participants provided consent and received gift cards for participating.

### 2.3 Procedures

Physicians were recruited at each site through information sessions and individual meetings to explain procedures and complete consent documents and baseline surveys. Patient recruitment procedures were designed to enroll similar patient numbers across physicians and across patient race (i.e., stratified by physician and race). Research staff identified eligible patients by examining upcoming appointments for participating physicians. Clinic staff informed eligible patients of the study and assessed interest. Research staff met with interested patients to explain the study, obtain consent, and administer baseline surveys. Then, research staff tracked participating patients for up to 2 years to identify those who had a scheduled appointment with a participating physician and were potentially eligible for an available therapeutic clinical trial, indicating there may be a trial discussion and invitation. These identified patients were asked to participate in the randomized controlled trial. This involved completing surveys before and after clinical interactions with a participating physician, being randomized to receive the communication intervention or usual care, and having their interaction with the physician video recorded. All surveys were completed using Qualtrics© survey software. Qualtrics randomly assigned patients to either the usual care or intervention group (1:1). Patients randomized to usual care participated in all study procedures, but did not receive the communication intervention. At the end of the two‐year period, research staff examined patient medical records to determine whether patients had received an invitation to participate in a therapeutic clinical trial.

#### 2.3.1 Patient intervention

The intervention was a paper brochure with attitude and communication components. The brochure included text and graphics specifically designed to portray inclusion of Black men (e.g., two of three photos in the brochure were of Black men). The attitude component was based on the Common Ingroup Identity Model,^26^ which posits that a sense of common identity or purpose increases cooperation and trust among individuals from different social backgrounds. The brochure attempted to create this identity by explaining that patients and physicians have equally important roles and should work together as a team to provide the best care for the patient's cancer. The communication component was a Question Prompt List (QPL) which included a list of questions related to clinical trials. QPLs have been used in several settings to improve clinical communication by encouraging patients to participate actively during medical visits, for example, by asking questions and stating their concerns.^27^, ^28^, ^29^ The QPL for PACCT was adapted from two existing QPLs with demonstrated effectiveness in similar populations and settings,^30^, ^31^ and pilot‐tested for acceptability with Black and White men. Research staff handed the brochure to patients in person and invited them to read through it, explaining briefly that patients might find it helpful during the clinic visit, especially if they discuss a clinical trial with their doctor. Research assistants were trained to encourage patients to ask questions during clinic visits, and to avoid answering questions or discussing trials.

### 2.4 Measures

The primary outcome for the prospective observational study was potential *eligibility* for an available therapeutic clinical trial (yes/no). Research assistants determined potential eligibility by reviewing medical records of patients with an upcoming appointment to see a participating physician, and matching their recent clinical status to eligibility requirements for available prostate cancer therapeutic clinical trials.

The outcomes for the prospective RCT were *communication quality* and *invitation* to participate in a specific therapeutic clinical trial. *Communication quality* during video‐recorded clinic visits was measured by patient self‐reports and observer ratings of patient active participation and physician patient‐centered communication (PCC). Patients and trained observers rated patient active participation using a measure assessing the extent to which patients engaged in each of seven behaviors, such as asking the doctor to explain treatments in greater detail.^31^ They rated physician PCC using a measure assessing the extent to which physicians engaged in each of the 12 behaviors. This measure has three subscales: informativeness (e.g., “doctor was very informative about patient's health”), supportiveness (e.g., “doctor made patient feel completely at ease”), and partnership‐building (e.g., “doctor asked for patient's thoughts about their health”).^31^, ^32^ Patients completed measures following their clinic visits. Observers were trained to observe the video‐recorded interactions and apply the measures. *Invitation* to a participate in a clinical trial was assessed by a single “yes/no” item determined by a review of patient medical records at the end of study enrollment.

Patient baseline measures included self‐reported socio‐demographic characteristics (race, age, income, education, health literacy^33^) and perceived health status.^34^

### 2.5 Sample size and statistical methods

For the prospective observational study, univariable and multivariable logistic mixed‐effects models with institution as a random effect were used to explore factors associated with potential trial eligibility. These factors included sociodemographic characteristics (race, age, education, income, health literacy) and perceived health status.

For the prospective intervention study, the planned study sample size was 16 patients per physician and 24 physicians (i.e., 384 patients) using the person‐level multi‐site/block trial design within Optimal Design,^35^ with the aim of achieving more than 95% power to detect a medium effect size of 0.5 at a 5% error. We further estimated that 169 eligible patients would have a video‐recorded trial discussion, producing at least 80% power to detect a medium effect size of 0.5 at a 5% error. However, the final sample for the prospective intervention study was small, and thus underpowered. Consequently, all statistical analyses were descriptive; no inferential analyses were conducted. Patient‐ and observer‐reported communication outcomes were summarized with means and standard deviations (SD) by study arm and race. Invitations to participate were summarized with counts and percentages by study arm and race.

## 3 RESULTS

### 3.1 Characteristics of study participants

**FIGURE 1:**
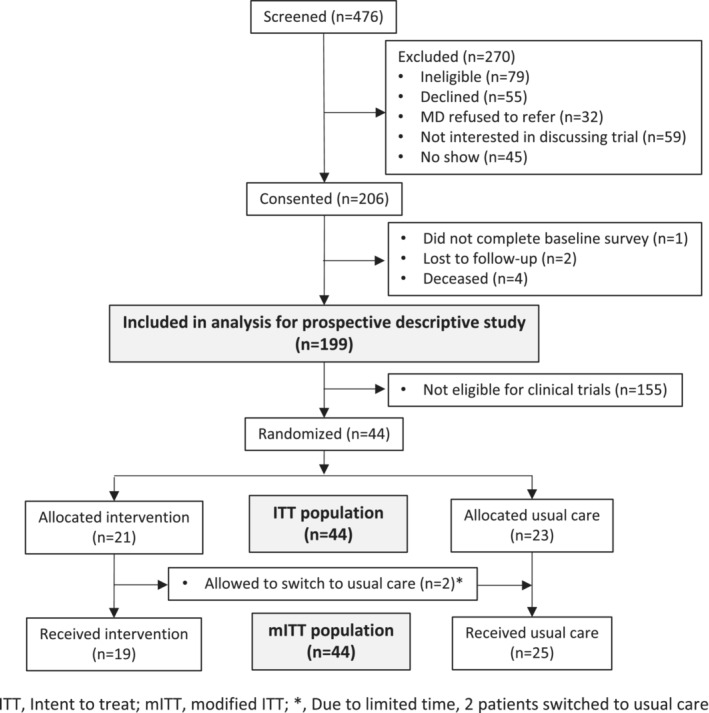
PACCT phase 1 participant CONSORT diagram.

Figure 1 shows patient participant flow and reasons for exclusions. During the study period (November 2016–January 2019), 476 patients of 18 physicians (10 at KCI, 8 at SKCCC) were screened. Of these, 156 (32.8%) patients were excluded because they were found to be ineligible (*n* = 79), their physician refused to refer them (*n* = 32), or they did not appear for their appointment (*n* = 45). Of the remaining 320 patients, 114 (35.6%) actively or passively declined, and 206 (64.4%) consented. Seven consented participants were excluded because they did not complete baseline surveys, were lost to follow‐up, or died. Table 1 provides baseline characteristics of patients included in the observational study (*n* = 199). Of these, 44 (22.1%) were found to be eligible for an available trial and were randomly assigned to either intervention or usual care in the intervention study. Of the intent‐to‐treat (ITT) population, 21 (47.7%) were randomized to the intervention group; two did not receive the intervention because they did not have time to complete it prior to their physician visit and were switched to the usual care group. Thus, in the modified intent‐to‐treat (mITT) sample (*n* = 44), 19 patients were in the intervention group and 25 in the usual care group. These patients were seen by a total of nine physicians, with usual care patients seen by eight physicians and intervention patients seen by six physicians. Table 1 provides baseline characteristics of these 44 participants by study arm. Of note, patient baseline characteristics were comparable between the two groups, although health literacy and income were higher in the usual care group.

**TABLE 1:** Patient baseline characteristics.

	Prospective observational study	Prospective intervention study		
	All (*n* = 199)	All (*n* = 44)	Usual care (*n* = 25)	Intervention (*n* = 19)
Race ‐ no. (%)				
Black	91 (45.7)	20 (45.5)	10 (40.0)	10 (52.6)
White	108 (54.3)	24 (54.5)	15 (60.0)	9 (47.4)
Age at consent ‐ mean (SD)	65.61 (8.38)	66.52 (8.50)	66.80 (8.03)	66.16 (9.30)
Education level‐ no. (%)				
Less than HS/High School/GED	64 (32.2)	14 (31.8)	8 (32.0)	6 (31.6)
Some College/College Degree	80 (40.2)	19 (43.2)	10 (40.0)	9 (47.4)
Graduate/Professional Degree	55 (27.6)	11 (25.0)	7 (28.0)	4 (21.1)
Household income ‐ no. (%)				
Less than $40,000	56 (29.6)	8 (19.5)	4 (16.7)	4 (23.5)
$40,000 ‐ $79,999	51 (27.0)	14 (34.1)	7 (29.2)	7 (41.2)
$80,000 or more	82 (43.4)	19 (46.3)	13 (54.2)	6 (35.3)
Missing	10	3	1	2
Perceived health status‐ mean (SD)	1.54 (0.53)	1.61 (0.52)	1.57 (0.48)	1.67 (0.57)
Missing	2	0	0	0
Health literacy ‐ mean (SD)	4.01 (1.01)	3.89 (0.95)	4.05 (0.89)	3.67 (0.99)
Missing		0	0	0
Study Site ‐ no. (%)				
Karmanos Cancer Institute	109 (54.8)	30 (68.2)	15 (60)	15 (78.9)
Sidney Kimmel Comprehensive Cancer Center	90 (45.2)	14 (31.8)	10 (40)	4 (21.1)

Abbreviation: SD, standard deviation.

### 3.2 Observational study results

Only 22.1% (*n* = 44) of study participants were potentially eligible for an available therapeutic trial during the study period. The only characteristic associated with eligibility was income in the multivariable analysis; patients with higher incomes were more often eligible than those with lower incomes (25.2% [=33/131] vs. 14.5% [=8/55]; Table 2). Compared to patients with incomes of <$40,000, patients with incomes of $40,000–$79,999 were more often eligible for a clinical trial (28.0% vs. 14.5%; adjusted OR, 4.40 [SD, 1.81]), as were those with incomes of >$80,000 (23.5% vs. 14.5%; adjusted OR, 6.06 [SD, 1.97]).

**TABLE 2:** Univariable and multivariable logistic mixed‐effects regression analyses of factors associated with eligibility (eligible vs. ineligible, ineligible as reference).

	Univariable (*n* = 44)		Multivariable (*n* = 41)	
	Eligible patients/Total (%)	OR (SE)	Eligible patients/Total (%)	OR (SE)
Race				
Black	20/91 (22.0)	Ref.	19/85 (22.4)	Ref.
White	24/108 (22.2)	1.07 (1.42)	22/101 (21.8)	0.65 (1.56)
Age at consent	44/199 (22.1)	1.02 (1.02)	41/186 (22.0)	1.03 (1.03)
Education level				
Less than HS/High School/GED	14/64 (21.9)	Ref.	14/58 (24.1)	Ref.
Some College/College Degree	19/80 (23.8)	1.20 (1.51)	16/74 (21.6)	0.64 (1.65)
Graduate/Professional Degree	11/55 (20.0)	0.99 (1.61)	11/54 (20.4)	0.50 (1.84)
Household income				
Less than $40,000	8/56 (14.3)	Ref.	8/55 (14.5)	Ref.
$40,000–$79,000	14/51 (27.5)	2.75 (1.67)	14/50 (28.0)	4.40 (1.81)
Over $80,000	19/82 (23.2)	2.36 (1.64)	19/81 (23.5)	6.06 (1.97)
Perceived health status	44/197 (22.3)	1.35 (1.36)	41/186 (22.0)	1.82 (1.44)
Health literacy	44/198 (22.2)	0.88 (1.18)	41/186 (22.0)	0.84 (1.24)

*Note*: Eligible Patients/Total (%), numbers of events (eligible patients) and patients, and percentage of eligible patients; OR, odds ratio; SE, standard error.

### 3.3 Intervention study results

Mean levels of patient active participation and physician patient‐centered communication were similar in the intervention and usual care groups (Table 3), and most were also similar across racial groups. However, Black intervention patients reported somewhat higher active participation than those in the usual care group (Intervention vs. Usual care; mean [SD], 4.14 [1.03] vs. 3.74 [0.99]), whereas White intervention patients reported somewhat lower active participation than those in the usual care group (4.11 [0.40] vs. 4.43 [0.62]). However, wide variation around these means preclude conclusions about reliable differences across these groups.

**TABLE 3:** Patient‐ and observer‐reported communication outcomes by study arm.

	Communication outcomes—mean (SD)					
	Patient‐reported			Observer‐reported		
	All (*n* = 44)	Usual care (*n* = 25)	Intervention (*n* = 19)	All (*n* = 44)	Usual care (*n* = 25)	Intervention (*n* = 19)
Pat active participation	4.14 (0.60)	4.14 (0.64)	4.13 (0.56)	2.79 (1.03)	2.89 (0.88)	2.66 (1.19)
Black	3.94 (1.04)	3.74 (0.99)	4.14 (1.03)	2.56 (1.50)	2.70 (1.59)	2.43 (1.46)
White	4.30 (0.59)	4.43 (0.62)	4.11 (0.40)	2.99 (1.37)	3.01 (1.05)	2.95 (1.98)
Missing	1	1	0	3	2	1
Physician PCC: Information‐giving	4.50 (0.54)	4.57 (0.50)	4.41 (0.54)	4.16 (0.54)	4.21 (0.50)	4.11 (0.58)
Black	4.47 (0.85)	4.45 (0.82)	4.50 (0.87)	4.21 (0.91)	4.39 (0.61)	4.05 (1.02)
White	4.52 (0.67)	4.66 (0.62)	4.31 (0.62)	4.12 (0.64)	4.09 (0.69)	4.19 (0.43)
Missing	1	1	0	3	2	1
Physician PCC: Supportiveness	4.43 (0.43)	4.48 (0.45)	4.37 (0.42)	4.08 (0.59)	4.17 (0.64)	3.96 (0.53)
Black	4.44 (0.64)	4.38 (0.64)	4.50 (0.64)	3.93 (1.00)	4.03 (1.32)	3.85 (0.74)
White	4.42 (0.60)	4.55 (0.61)	4.22 (0.47)	4.20 (0.67)	4.27 (0.64)	4.09 (0.75)
Missing	1	1	0	3	2	1
Physician PCC: Partnership‐building	4.22 (0.59)	4.23 (0.70)	4.20 (0.47)	3.84 (0.49)	3.90 (0.46)	3.76 (0.53)
Black	4.12 (0.95)	4.08 (1.26)	4.17 (0.66)	3.76 (1.00)	3.89 (1.17)	3.65 (0.87)
White	4.29 (0.75)	4.34 (0.79)	4.22 (0.71)	3.91 (0.34)	3.91 (0.27)	3.91 (0.49)
Missing	1	1	0	3	2	1
Physician PCC: Total	4.38 (0.47)	4.43 (0.51)	4.32 (0.43)	4.03 (0.42)	4.09 (0.38)	3.94 (0.47)
Black	4.35 (0.74)	4.30 (0.85)	4.39 (0.64)	3.97 (0.82)	4.10 (0.81)	3.85 (0.78)
White	4.41 (0.64)	4.52 (0.64)	4.25 (0.58)	4.08 (0.38)	4.09 (0.39)	4.06 (0.37)
Missing	1	1	0	3	2	1

Abbreviations: PCC, patient‐centered communication; SD, standard deviation.

Regarding rates of invitation to participate in clinical trials, 73.7% of intervention participants compared to 60.0% of usual care participants received an invitation (Table 4). This difference in invitation rates by study arm was larger among Black men (Intervention vs. Usual care, 80.0% [=8/10] vs. 30.0% [=3/10]) than among White men (66.7% [=6/9] vs. 80.0% [=12/15]).

**TABLE 4:** Clinical trial invitation by study arm and patient race.

	Number of trial invitations/total patients (%)	
	Usual care	Intervention
Clinical trial invitation	15/25 (60.0)	14/19 (73.7)
Black	3/10 (30.0)	8/10 (80.0)
White	12/15 (80.0)	6/9 (66.7)

*Note*: Number of trial invitations/total patients (%), numbers of events (invitations) and patients, and percentage of invited patients.

## 4 DISCUSSION

Participation in cancer clinical trials is consistently and persistently low, reflecting inequitable access to high‐quality care. This study is among the first to examine multilevel enrollment barriers and to examine an intervention to increase enrollment among Black and White patients who are potentially eligible for an available therapeutic trial.

The first barrier we examined was eligibility, a system‐level barrier. Findings showed that less than a quarter of participants qualified for an available trial over the two‐year period they were enrolled in the study. This small proportion was surprising, given that the study participants were receiving care at major academic institutions and had a disease associated with many therapeutic trials. Also, despite research suggesting that trial participation is associated with age, race, and other sociodemographic characteristics,^4^, ^20^ the only characteristic associated with eligibility was income. Prior studies have found that higher socioeconomic status (SES) is associated with higher prostate cancer incidence, lower likelihood of presenting with metastatic disease at the time of diagnosis, and lower mortality rates of prostate cancer.^1^, ^36^, ^37^ These associations are presumably due to greater access to healthcare among those with higher SES. However, this is one of the very few studies to specifically examine the association between SES and clinical trial participation.^1^, ^38^ Future studies are needed to not only further document the extent to which SES affects clinical trial enrollment, but also test interventions to mitigate these effects. Interventions may include, for example, offering patients the ability to participate in some aspects of a trial in their local clinics (e.g., blood draws, scans); providing transportation to clinical trial research sites; and compensating patients for trial‐related time and expenses.

Together, these findings provide further evidence that the greatest barrier to enrollment may be restrictive eligibility criteria—most patients do not qualify.^39^ Thus, interventions targeting patients' attitudes and perception of trials may be misguided, and in fact, may cause backlash in the form of greater mistrust when patients learn the value of participation, only to discover they do not qualify. Several professional organizations have published recommendations for addressing inequitable enrollment.^8^, ^40^ Recommendations include, for example, designing and implementing trials with a specific focus on reducing barriers and enhancing equity, diversity, and inclusion (EDI); requiring training and a commitment to achieving EDI for investigators, sponsors, and policymakers, and shifting to a paradigm that prioritizes the interests and voices of the community. PACCT's protocol, for example, required recruiting an equal number of Black and White patients per physician, which required recruiters to make extra efforts to achieve equitable enrollment.^22^ Such strategies could improve inclusiveness, access and enrollment, thereby improving the generalizability of results and accelerating the pace of scientific progress.^39^, ^41^ Additionally, findings suggest that published enrollment rates should consider not only all patients, but also all *eligible* patients as a meaningful denominator, thus providing more accurate data on the sources of low enrollment and potential intervention targets.^42^

The second enrollment barrier we addressed was interpersonal: patient–physician communication quality in interactions with potentially eligible patients. This low‐cost, patient‐focused communication intervention was based on the assumption that, while patient–physician clinical communication is the most central and proximal influence on invitations to participate and actual trial participation, communication about trials may be infrequent or of low‐quality, especially for Black patients.^43^ The intervention was designed to encourage patients to participate actively in clinical interactions to improve communication quality and prompt invitations to participate. Although the study sample was very small and results must be interpreted with caution, the intervention showed improved outcomes, especially among Black participants, as we expected. Compared to usual care patients, Black intervention patients reported participating more actively in clinical interactions, such as by asking questions and stating concerns. While this finding is promising, questions remain about why White intervention patients reported *less* active participation. Perhaps of greater importance, both Black and White intervention patients received more trial invitations than usual care patients, and Black intervention patients received more invitations than White intervention patients.

This study should be interpreted within its limitations. The sample size was small due to the COVID‐19 pandemic disruptions,^24^, ^25^ which occurred at the beginning of Phase 2 of the study. Most clinical trials were paused and hospitals prohibited research staff from entering the clinics. Thus, this study includes only data from Phase 1, limiting originally‐planned analyses and suggesting caution in interpreting findings. We could not examine some planned outcomes, including patient decisions to participate and actual enrollment rates. We also could not test the effects of the physician‐focused communication intervention on outcomes. Thus, future research is needed in a larger sample to complete these analyses.

Additionally, the study included only Black and White men with one type of cancer, and was conducted at major academic centers, limiting the generalizability of the study. Future studies should include a broader population, such as patients receiving care at community cancer centers,^44^ where the two barriers addressed here may be more pronounced.

Despite these limitations, this study suggests ways to improve equitable clinical trial enrollment. Suggested strategies include broadening trial eligibility criteria to better reflect and include diverse patient populations; reporting trial enrollment rates not just among all people with cancer, but also among those potentially eligible for an available trial as a way to identify potential intervention targets; and implementing low‐cost communication interventions, such as the one described here—a low‐cost, highly scalable intervention that may improve patient‐provider communication and invitations to participate in clinical trials, and shows specific benefit for Black patients, who have been historically excluded from or mistreated in clinical trials.

## PRECIS

This multisite study addressed two barriers to enrollment of Black and White men in prostate cancer clinical trials. Findings showed that eligibility, a systemic barrier, is the biggest barrier, but that a communication intervention can improve interpersonal barriers including communication quality and trial invitation rates, especially for Black patients.

## AUTHOR CONTRIBUTIONS

**Susan Eggly:** Conceptualization (equal); data curation (equal); formal analysis (equal); funding acquisition (equal); investigation (equal); methodology (equal); project administration (equal); supervision (equal); writing – original draft (equal); writing – review and editing (equal). **Nicole Senft:** Conceptualization (equal); formal analysis (equal); methodology (equal); writing – review and editing (equal). **Seongho Kim:** Formal analysis (equal); methodology (equal); writing – review and editing (equal). **Elisabeth I Heath:** Conceptualization (equal); methodology (equal); writing – review and editing (equal). **Hyejeong Jang:** Formal analysis (equal); writing – review and editing (equal). **Tanina Foster:** Formal analysis (equal); project administration (equal); writing – review and editing (equal). **Fatmeh Baidoun:** Project administration (equal); writing – review and editing (equal). **Mark Manning:** Conceptualization (equal); formal analysis (equal); methodology (equal); writing – review and editing (equal). **Louis A. Penner:** Conceptualization (equal); formal analysis (equal); methodology (equal); writing – review and editing (equal). **Terrance Albrecht:** Conceptualization (equal); methodology (equal); writing – review and editing (equal). **Michael A Carducci:** Conceptualization (equal); project administration (equal); supervision (equal); writing – review and editing (equal). **Dina Lansey:** Conceptualization (equal); project administration (equal); writing – review and editing (equal). **Lauren Hamel:** Conceptualization (equal); formal analysis (equal); methodology (equal); writing – review and editing (equal).

## FUNDING INFORMATION

This research was supported by the National Institutes of Health/National Cancer Institute Grant No. R01CA200718 (S.E)

## CONFLICT OF INTEREST

The following authors report no conflict of interest: Susan Eggly, Nicole Senft, Seongho Kim, Tanina Foster Moore, Fatmeh Baidoun, Mark Manning, Louis Penner, Terrance Albrecht, Michael Carducci, Dina Lansey, and Lauren Hamel. Elisabeth Heath reports serving as consultant for Astellas Pharma and on the Ad Board and/or Speakers' Bureau for Astra Zeneca, Sanofi, and Bayer.

## Data Availability

The data that support the findings of this study are available on request from the corresponding author.
